# Phytochemicals of Apple Pomace as Prospect Bio-Fungicide Agents against Mycotoxigenic Fungal Species—In Vitro Experiments

**DOI:** 10.3390/toxins11060361

**Published:** 2019-06-20

**Authors:** Marta Oleszek, Łukasz Pecio, Solomiia Kozachok, Żaneta Lachowska-Filipiuk, Karolina Oszust, Magdalena Frąc

**Affiliations:** 1 Institute of Agrophysics, Polish Academy of Sciences, Doświadczalna 4, 20-290 Lublin, Poland; zaneta_lachowska@wp.pl (Ż.L.-F.); k.oszust@ipan.lublin.pl (K.O.); m.frac@ipan.lublin.pl (M.F.); 2 Institute of Soil Science and Plant Cultivation, State Research Institute, Czartoryskich 8, 24-100 Puławy, Poland; lpecio@iung.pulawy.pl (Ł.P.); skozachok@iung.pulawy.pl (S.K.); 3 I. Horbachevsky Ternopil National Medical University, Maidan Voli 1, 46001 Ternopil, Ukraine

**Keywords:** mycotoxins, *Fusarium* sp., *Botrytis* sp., apple pomace, phloridzin, quercetin glycosides, pinnatifidanoside D

## Abstract

The phytochemical constituents of apple waste were established as potential antifungal agents against four crops pathogens, specifically, *Botrytis* sp., *Fusarium oxysporum, Petriella setifera*, and *Neosartorya fischeri*. Crude, purified extracts and fractions of apple pomace were tested in vitro to evaluate their antifungal and antioxidant properties. The phytochemical constituents of the tested materials were mainly represented by phloridzin and quercetin derivatives, as well as previously undescribed in apples, monoterpene–pinnatifidanoside D. Its structure was confirmed by 1D- and 2D-nuclear magnetic resonance (NMR) spectroscopic analyses. The fraction containing quercetin pentosides possessed the highest antioxidant activity, while the strongest antifungal activity was exerted by a fraction containing phloridzin. Sugar moieties differentiated the antifungal activity of quercetin glycosides. Quercetin hexosides possessed stronger antifungal activity than quercetin pentosides.

## 1. Introduction

Billions of tons of agricultural waste are generated every year. A substantial part of them causes pollution problems, when they are not managed properly [1]. Apples are one of the crops with the largest annual production worldwide. Poland is one of the major producers of apples, with ca. 3.6 million metric tons of apples produced every year [2]. On the other hand, crop residues are a rich source of biologically active compounds and may become the important raw materials for obtaining various valuable by-products [3]. Apple pomace consists of apple skin, seeds, and flash, and represents about 25% of a fruit’s fresh weight [4]. The main bioactive compounds of apple processing by-products are, in particular, flavonoids (phloretin and quercetin glycosides, flavone derivatives and catechins) as well as organic acids [5,6]. Their applications have been addressed to exploit antioxidant and pharmacological properties. Kołodziejczyk et al. [7] stated that polyphenols from industrial pomace were good antimicrobial agents against human pathogens such as *Salmonella* spp., *Escherichia coli* and *Listeria* spp. Two main flavonoids of apple, phloretin and quercetin, have previously been isolated from apple fruits and tested against various fungi [8,9]. However, to the best of our knowledge, there is a lack of evidence on the usage of phytochemicals of industrial pomaces, particularly from apples, as natural bio-pesticides or bio-fungicides for organic farming [1]. There is a wide variety of chemically synthetized pesticides [10], but their application leads to a resistance and causes the selection of less-sensitive isolates [11]. Resistance to antimicrobial agents is consistently increasing and becoming a global problem. Moreover, many industrial fungicides are harmful to humans and detrimental to animal health [9]. For this reason, there is an urgent need to find new or more efficient, safe and ecologically friendly antifungal agents, especially against toxigenic fungi, that could be applied in organic farming [12].

A set of the following fungi: *Botrytis* sp., *Fusarium oxysporum, Petriella setifera* and *Neosartorya fischeri* is posing a worldwide threat in farming, gardening and food processing. *Fusarium* species may cause plant diseases of both underground and aboveground parts and can produce mycotoxins [10,13]. *Botrytis* sp. is an important pathogen in many economically important crops [14]. Additionally, *P. setifera* was classified as a potential plant pathogen [15]. The economic importance of this pathogen is connected with forest and especially with oak trees [16]. The other relevant magnitude of *Petriella* sp. is participation in wood decay as soft rot fungi or sometimes as brown rot fungi [17,18]. On the other hand, contamination by heat-resistant fungi such as *N. fischeri* is a major problem for the fruit-processing industry in many countries, due to mycotoxins such as verruculogen and fumitremorgins [19].

Data from the literature proved that flavonoids participate in the reaction against pathogen, both as components of plants tissues, but also when they are applied externally [12,20,21,22]. Sanzani et al. [20] showed that quercetin is very effective in reduction of *Penicilium expansum* growth and patulin accumulation in stored apples. The inhibited effect of low concentration of quercetin and rutin was observed also in vitro on *F. oxysporum* [21]. Parvez et al. [23] proved inhibitory effect of quercetin-3-O-glucoside (isoquercitrin) and quercetin-3-methyl ether, as well as its glycosides, on the conidial germination of *Neurospora crassa*.

Therefore, the aim of the presented study was to determine the antifungal and antioxidant activity of apple pomace’s crude and purified extracts, chromatographic fractions and to evaluate their suitability as a source of natural bio-fungicides against *Botrytis* sp., *Fusarium oxysporum, Petriella setifera* and *Neosartorya fischeri*. For antifungal activity determination, a new, fast and simple instrumental method utilising BIOLOG MT2 Plates^®^ was applied and optimised in the place of conventional hole-plate method [10]. Phytochemical constituents of the studied object were established by means of ultra high performance liquid chromatography-photodiode array detection-mass spectrometry (UHPLC-PDA-MS) analysis. Furthermore, for the first time, the undescribed constituent of apple-monoterpene pinnatifidanoside D was isolated and structurally elucidated.

## 2. Results and Discussion

Among all identified compounds, hyperoside, quercitrin and phloridzin were the most abundant in crude extract (CE) and purified extract (PE); (Table 1, Figure S1–S6. These results were in accordance with previous study [24,25,26]. Additionally, other flavonoids such as isoquercetin, rutin, reynoutrin, quercetin-3-O-pentosyls, avicularin, quercitrin and quercetin were determined. Furthermore, one monoterpene, not detected previously in apples-pinnatifidanoside D was isolated and structurally elucidated by extensive 1D and 2D nuclear magnetic resonance (NMR) spectroscopic analyses (Figure S7–S15). Characteristic data of this compound are as follows: pinnatifidanoside D: white amorphous solid; ultraviolet (UV) Λ_max_ (UPLC-PDA) 240 nm; electrospray ionization-in-source collision-induced dissociation mass spectrometry (ESI-isCID MS) (% of base peak) *m/z* 541 [M + Na]^+^ (22), 519 [M + H]^+^ (16), 387 [M – 132 + H]^+^ (27), 225 [M – 132 – 162 + H]^+^ (13), 207 [M – 132 – 162 – 18 + H]^+^ (100), 189 [M – 132 – 162 – 2 × 18 + H]^+^ (13), 161 (11), 149 (17), 123 (37); ^1^H-NMR (500 MHz, MeOH-d_4_), δ_H_ 5.89 (1H, t-like, J = 1.3 Hz, H-4), 5.85 (2H, m, H-7, 8), 4.43 (1H, qd, J = 6.4, 1.9 Hz, H-9), 4.35 (1H, d, J = 7.8 Hz, H-1′), 4.28 (1H, d, J = 7.5 Hz, H-1′’), 4.06 (1H, dd, J = 11.3, 1.8 Hz, H-6a’), 3.86 (1H, dd, J = 11.5, 5.3 Hz, H-5a’’), 3.69 (1H, dd, J = 11.3, 4.8 Hz, H-6b’), 3.49 (1H, ddd, J = 10.1, 8.7, 5.3 Hz, H-4′’), 3.35 (2H, m, H-4′, 5′), 3.34 (1H, m, H-3′), 3.31 (1H, t, J = 8.8 Hz, H-3′’), 3.22 (1H, dd, J = 9.0, 7.5 Hz, H-2′’), 3.18 (1H, t-*like*, J = 8.4 Hz, H-2′), 3.18 (1H, t, J = 10.8 Hz, H-5b’’), 2.51 (1H, d, J = 16.9 Hz, H-2a), 2.16 (1H, d, J = 16.9 Hz, H-2b), 1.92 (3H, d, J = 1.4 Hz, H-13), 1.29 (3H, d, J = 6.4 Hz, H-10), 1.04 (3H, s, H-11), 1.03 (3H, s, H-12); ^13^C-NMR (125 MHz, MeOH-d_4_), δ_C_ 201.2 (C-3), 167.2 (C-5), 134.9 (C-8), 131.7 (C-7), 127.2 (C-4), 105.6 (C-1′’), 102.6 (C-1′), 80.0 (C-6), 77.9 (C-3′), 77.7 (C-3′’), 76.9 (C-9), 76.8 (C-5′), 75.2 (C-2′), 74.8 (C-2′’), 71.3 (C-4′), 71.2 (C-4′’), 69.8 (C-6′), 66.9 (C-5′’), 50.8 (C-2), 42.5 (C-1), 24.7 (C-12), 23.5 (C-11), 19.7 (C-13).

In turn, pinnatifidanoside D was the main component of fraction 1 (F1). This compound has been isolated for the first time from *Crataegus pinnatifida* [27]. Li et al. [27] stated also that pinnatifidanoside D exhibited small antiplatelet aggregation activity.

The LH20 fractions F2 and F3 contained many unknown compounds of various structures. Their identification was left for separate investigation. Analysing the UV-spectres, tandem mass spectrometry (MS/MS) fragmentation pattern and literature data allows us to identify the main components of the F4, F5 and F6. The major compound of F4 was phloridzin, flavonoid belonging to chalcones group. Fractions F5 and F6 consisted of quercetin derivatives, while F5 contained mostly quercetin with hexoside moieties, and F6 included mainly quercetin with pentoside moieties (Table 1).

The presence of phloretin and quercetin derivatives (glucoside, galactoside, xyloside, arabinoside, rhamnoside) in apples and their residues, particularly skins, has already been well recognised and confirms the results of the present study [24,25,28]. Moreover, many previous reports have also shown the presence of procyanidin B, epicatechins and chlorogenic acid as the major phenolic compounds in apple [29,30,31,32]. Tested crude extract did not contain catechins, probably because they are sensitive to oxidation by heat and light [33]. For the further investigation of antioxidants and antifungal activity, the LH20 fractions with established composition (F1, F4–F6) were selected.

The results of reducing power and radical-scavenging activity showed that apple pomace contained strong antioxidants. The antioxidant activity of the CE was low, due to the high content of the polar fraction (PF) containing mainly simple sugars, which do not exhibit antioxidant properties (Table 2). Nevertheless, the PE presented much higher values of the tested parameters. When it comes to LH20 fractions, values of EC_50_ and IC_50_ decreased along with subsequent fraction number. At the same time, F5 and F6 were not significantly different in terms of IC_50_ value of radical scavenging activity. The antioxidant activity depends on the structure of compounds, primarily the presence of hydroxyl, 4-oxo and catechol group as well as 2–3 double bond [34]. For this reason, the F4, containing mainly phloridzin, exhibited lower antioxidant properties (higher EC_50_ and IC_50_) than F5 and F6, which included quercetin derivatives. Quercetin is known as a strong antioxidant, mainly due to the presence of catechol group in ring B [35].

**Figure 1:**
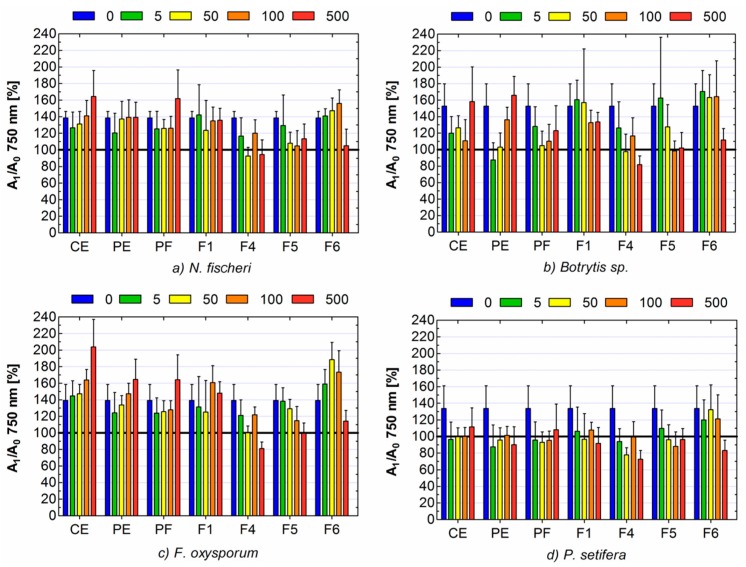
The impact of the apple pomace crude extract, purified extract and its fractions on the growth of fungi: (**a**) *Neosartoria fischeri*, (**b**) *Botrytis* sp., (**c**) *Fusarium oxysporum*, (**d**) *Petriella setifera*. The concentrations of solutions was 0, 5, 50, 100, 500 μL ml^−1^. A1/A0—the ratio of absorbance for tested samples with fungi and absorbance for adequate control (sample alone, without fungi), CE—crude extract, PE—purified extract, PF—polar fraction and F—LH20 subfraction. Bars represent the mean of 24 replicates ± standard deviation.

Microbiological assays showed that apple pomace contained compounds with antifungal activity (Figure 1). In the case of *P. setifera* all tested formulations caused inhibition of the mycelium growth even at quite low doses. The exception was F6, which exhibited antifungal properties only at the highest concentration of 500 μg mL^−1^. The CE of apple pomace caused also inhibition of the growth of *Botrytis* sp. at concentration in the range of 5–100 μg mL^−1^, it stimulated the growth of *F. Oxysporum* and did not influence significantly the growth of *N. fischeri*.

Generally, the purification of crude extract increased its antifungal activity or weakened its stimulating effect, though the differences between CE and PE was not significant. Among all fractions, F1 showed no significant influence on *N. fischeri*, *Botrytis* sp., *F. oxysporum*, even regardless of the dose. Its effect on *P. setifera* was negative, but it was also independent from the concentration. On the contrary, F4 exhibited the strongest activity against all fungal strains. Moreover, it can be noticed, that A_1_/A_0_ absorbance ratios in the case of higher concentrations of F4 were below 100% (Figure 1). It means that absorbance for the control (the solution of tested substance without fungi) was higher than absorbance for tested sample (solution of tested substance with fungi). It can be supposed that such a low absorbance ratio was due to the fact that fungi intensively utilized the tested sample. Consequently, the concentration of the tested sample was decreased and its influence on the value of absorbance of the tested sample was reduced. To confirm this supposition, the ratio of absorbance at 490 nm and 750 nm was measured (A_490_/A_750_); (Table 3).

The absorbance at 490 nm reflects the respiration rate, so also substrate use, while the value of absorbance at 750 nm informs us about biomass/turbidity production (growth pattern) [36]. According to the above, A_490_/A_750_ ratio much higher than 1 indicates stressful metabolic situation, when a small biomass (low absorbance at 750 nm) yielding high respiration rates (high absorbance at 490 nm). The highest values of A_490_/A_750_ were noted for the highest concentration of F4: 1.44, 1.20 and 1.26 for *F. oxysporum*, *Botrytis* sp., *P. setifera*, respectively (Table 3). In the case of *N. fischeri*, this ratio was 1.03, and no significant drop of A_1_/A_0_ below 100% was observed (Figure 1a).

The main compound of F4—phloridzin plays a major role in apple in the resistance to fungal infection. It is metabolized to phloretin and then, to the next oxidation products such as *o*-quinone, which are fungitoxic [37,38,39]. Antifungal activity of phloridzin and its aglycone, phloretin, was previously described [8,40]. The first report on the antifungal activity of phloretin against plant pathogenic fungi was done by Shim et al. [8], who investigated the influence of phloretin isolated from apple against *B. cinerea*, *F. oxysporum* and five other fungi. The results showed that phloretin could be used as biopesticide for control of rice blast as well as tomato late blight.

The F5 and F6 consisted of quercetin derivatives (Table 1). Quercetin, similarly to phloretin, affects the resistance of plants to fungal diseases. Lee et al. [41] observed the increase in concentration of quercetin glycosides in onion infected by *F. oxysporum.* Sanzani et al. [20] stated that quercetin in apple is responsible for the resistance on *P. expansum* and inhibition of patulin synthesis. For this reason, it can be considered as a natural compound to be used as alternative strategy to chemical fungicides in post-harvest control of *P. expansum* infections [9].

Despite the fact that both F5 and F6 contained quercetin glycosides, they differed meaningfully in their antifungal properties. F5 containing mostly quercetin hexosides almost completely inhibited the growth of *N. fischeri*, *Botrytis* sp., *P. setifera* at the concentration of 100 μg mL^−1^. No growth of *F. oxysporum* was observed only at the highest concentration of 500 μg mL^−1^. F6 including mostly quercetin pentosides, rather, stimulated the fungal growth until the dose of 100 μg mL^−1^, although in the case of *N. fischeri*, *Botrytis* sp. and *P. setifera* the effect was not significant. Antifungal activity of F6 was detected only at the highest dose in the case of all tested isolates. The difference in antifungal properties between quercetin hexosides and pentosides is not entirely clear. Dissimilarity in the antifungal action of various quercetin glycosides was stated previously [23]. The authors reported that quercetin-3-*O*-glucoside (isoquercitin) was the only non-methylated flavonoid to inhibit conidial germination of *Arabidopsis thaliana* and *Neurospora crassa*. Among the tested quercetin derivatives were: quercetin-3-O-galactoside (hyperoside), quercetin-3-O-arabinoside (avicularin), quercetin-3-O-rhamnoside and quercetin. The antifungal effect was not noted for those quercetin derivatives, despite seemingly being very similar chemical structure. Various actions in spite of the same aglycone may result from the fact that glycosides rarely were metabolized to aglycone, but very often to higher molecules by glycosylation, sulfonation or methylation [42,43]. Simultaneously, a wide range of metabolic activity towards flavonoids exists in different fungal strains [43]. Generally, it is widely known that bioactive action of compounds depends on their structures and the bioactivity of flavonoids is ascribed to their aglycone moiety [43,44]. Nonetheless, there is a plethora of studies reporting a stronger antifungal effect of substituted flavonoids than unsubstituted ones [23,44]. The result of the present study showed also that antifungal activity do not always go hand in hand with antioxidant activity, because both properties depend on different structural conditions of the compounds.

Gauthier et al. [45] reported that antifungal activity of flavonoids directly resulted from their ability to combine irreversibly with nucleophilic aminoacids in fungal proteins. Moreover, they inhibit proteins to form several hydrogen and ionic bonds and disturb three-dimensional structure transporters [12]. As was mentioned by Lourenço et al. [12], flavonoids may be of interest in the agriculture as they can enhance the activity of pesticides, as well as reverse resistance to synthetic preparations.

In conclusion, the results of the study showed that apple pomace could be a good source of natural bio-fungicides, due to inhibition of mycotoxigenic fungal growth. The crude extract, contained mainly polar compounds like sugars, as well as phloridzin and quercetin glycosides, but also monoterpene—pinnatifidanoside D, which was for the first time isolated from apple waste. The effect of crude extract and its fractions was similar towards all tested fungi species. The strongest antifungal activity was exhibited by fraction F4 containing phloridzin, while the highest antioxidant activity was showed by fraction F6 containing mainly quercetin pentosides. Sugar moiety significantly determines the antifungal activity of quercetin glycosides. Despite the same aglycone of constituents of F5 and F6, they differed in their antifungal properties. Both antioxidant and antifungal activities of fraction F1, containing pinnatifidanoside D, were rather low. That means that the screening of proper bioactivity for this poorly studied compound is required. The antifungal and antioxidant effects did not go hand in hand, probably because of the differences in structural conditions of the compounds determining these properties.

## 3. Materials and Methods

### 3.1. Materials

#### 3.1.1. Apple Pomace

The tested material—apple pomace—was supplied from local apple juice-processing factory. Raw material was lyophilised, powdered and subjected to extraction. The CE of apple pomace, PE and F1-F6 obtained by gel-filtration of the PE using Sephadex LH20 were tested as potential bio-fungicides against four selected fungi: *Botrytis* sp., *F. oxysporum*, *P. setifera* and *N. fischeri.*

#### 3.1.2. Fungal Strains and Culture Conditions

Four fungal isolates were taken for the experiments. Three isolates (*Botrytis* sp., *F. oxysporum*, *P. setifera*) were selected from the Laboratory of Molecular and Environmental Microbiology (LMEM), Institute of Agrophysics, Polish Academy of Sciences (Lublin, Poland) and one strain (*N. fischeri* G90/14) was obtained from the National Institute of Technology and Evaluation, Biological Research Centre, NITE (NBRC). *Botrytis sp.* G669/16 and *F. oxysporum* G648/16 were isolated from strawberry, while *P. setifera* G11/16 was obtained from compost for agricultural usage. All strains were cultured on 90 mm Petri dishes with potato dextrose agar (PDA) at 27 °C for 5 days in the dark prior to DNA isolation. The isolates from (LMEM) collection were identified on the basis of the D2 domain of large-subunit ribosomal DNA (D2 LSU rDNA) or internal transcribed spacer 1 rRNA (ITS1) sequencing (Thermo Fisher Scientific, United States) according to methodology [18,46]. The following universal primers D2LSU2_F (5′-AGA CCG ATA GCG AAC AAG-3′) and D2LSU2_R (5′-CTT GGT CCG TGT TTC AAG-3′) [18] and ITS1 (5′-TCC GTA GGT GAA CCT GCG G-3′) and ITS2 (5′-GCT GCG TTC TTC ATC GAT GC-3′) [47], were used for D2 LSU and ITS1, respectively. The run was performed in final volume of 20 µL using a Veriti 96-Well Fast Thermal Cycler (Applied Biosystems, Foster City, CA, USA) in the following conditions: 95 °C for 10 min, then 35 cycles at 95 °C for 15 s, 53 °C for 20 s and 72 °C for 20 s and followed by a final step at 72 °C for 5 min. Nucleotide sequences of the strains were deposited in the National Centre for Biotechnology Information (NCBI) under the following accession numbers: KX639294.1, KX639319.1, MG594608, respectively. The fourth isolate *N. fischeri* obtained from the NBRC collection was designed as isolate number NBRC 31895. Prior to antifungal analysis, strains were cultured for 14 days on 90 mm Petri dishes with potato dextrose agar (PDA) in the dark at 27 °C to obtain conidial spores. Next, the cultures were harvested into sterile BagPage^®^ membrane filters containing IF-FF liquid and processed using an Ultra Turax IKA^®^ homogenizer for 30 s and then filtered to extricate spores. Spores of each strain were used to set up 75% transmittance inoculum measured with a turbidimeter (Biolog^®^) to serve as inoculum for 96-well MT2 microplates (Biolog^®^) to analyze antifungal activity.

### 3.2. Extract Preparation and Fractionation

The CE was obtained according to the method described by Oleszek and Krzemińska [48]. Briefly, 30 g of powdered, dried material was defatted with chloroform in a Soxhlet apparatus, and then extracted (3 × 300 mL, 20 min. each) by sonication with 70% aq. MeOH at room temperature in the dark place. The CE was concentrated using the rotary evaporator under reduced pressure (at 40 °C) and freeze-dried to yield 9.95 g (33.17% of the dry plant material).

In the next step, the CE was dissolved in Milli-Q water and purified on a short self-packed RP-C_18_ column (60 mm × 100 mm, 75 μm, Cosmosil 75C18-PREP). The polar fraction (PF) of CE, included sugars and simple organic acids and was eluted by acidified water (0.1% formic acid, *v*/*v*), while purified extract (PE) containing plant specific metabolites was eluted with methanol-water (95:5, *v*/*v*) solution. Obtained solutions were evaporated, suspended in *t*-butanol-water solution and freeze-dried to obtain 9.11 g of PF, and 0.84 g of PE. Afterwards, the PE was fractionated on a Sephadex LH-20 (40–120 μm) glass column (95 cm × 3.2 cm) and connected to a Gilson prep-HPLC (high-performance liquid chromatography) system with ELS™ II detector. The separation was achieved by the flow of acidified 95% MeOH (0.1% formic acid) at a flow rate of 2.4 mL min^−1^ [49]. Six LH-20 fractions were collected according to the ELS chromatogram, evaporated and freeze-dried to obtain: F1 (0.18 g), F2 (0.17 g), F3 (0.01 g), F4 (0.03 g), F5 (0.06 g), F6 (0.03 g); (Figure S16). The fractions were kept at freezer for further analysis.

### 3.3. Phytochemical Analysis

#### 3.3.1. Identification and Quantification of Individual Compounds in Crude, Purified Extracts and Its Fractions

The CE and PE, as well as LH20 fractions (F1-F6) were analysed by Waters ACQUITY UPLC system (Waters Corp., Milford, MA, USA) equipped with a binary pump system, sample manager, column manager, and MS and PDA detectors (Waters Corp). For acquisition and data processing, Waters MassLynx software v.4.1 was used. The separation was carried out on the ACQUITY UPLC BEH C_18_ column (100 mm × 2.1 mm, 1.7 μm, Waters Corp., Milford, MA, USA) at temperature of 40 °C and flow rate adjusted to 400 μL min^−1^. The injection volume of the sample was 2.5 μL. The mobile phase was composed of 0.1% (v/v) formic acid in Milli-Q water (solvent A) and acetonitrile with 0.1% (*v*/*v*) formic acid (solvent B). Gradient program was as follows: 0–1.5 min, 10% B; 1.5–15.0 min, 10–25% B; 15.0–15.10 min, 25–100% B; 15.1–16.6 min, 100% B; 16.6–16.7 min, 100–10% B; 16.7–20.0 min, 10% B. The MS analyses were carried out on a Waters ACQUITY TQD (tandem quadrupole detector) (Waters Corp) equipped with a Z-spray electrospray interface. The parameters for ESI source were: capillary voltage 2.8 kV, cone voltage 45 V, desolvation gas N_2_ 800 L h^−1^, cone gas N_2_ 100 L h^−1^, source temp. 140 °C, desolvation temp. 350 °C.

Peaks were assigned based on their retention times, mass to charge ratio (*m*/*z*), and ESI-MS/MS fragmentation pattern, as well as their comparison to the previously isolated standards, Department of Biochemistry and Crop Quality, IUNG. The individual compounds were quantified by the external standard method using the calibration curves of pinnatifidanoside D (240 nm, 0.010–0.482 µmol/mL), rutin (355 nm, 0.008–0.410 µmol/mL) for quercetin glycosides calculation, with five different concentration levels (*R*^2^ ranged between 0.9923 and 0.9997). The molar concentration was plotted against peak area. Due to the lack of a phloridzin standard, structurally similar (αS)-4′-O-β-D-glucopyranosyl-α,2′,4-trihydroxydihydrochalcone with 436 MW, previously isolated from lentil root [50], was used for constructing the calibration curve at 284 nm (0.002–0.401 µmol/mL).

#### 3.3.2. Isolation Process of Pinnatifidanoside D

LH20 F1 was subjected to semi-preparative HPLC, equipped with a Gilson 321 pump, a Gilson GX-271 liquid handler with a 2 mL sample loop and a Gilson Prep ELS™ II detector. Pinnatifidanoside D (9.1 mg) was isolated in an isocratic mode using CH_3_CN:H_2_O:FA (13:87:0.1, *v*/*v*), at 4 mL min^−1^, on Atlantis Prep T3 at 40 °C.

#### 3.3.3. Nuclear Magnetic Resonance (NMR) Analysis

The pure isolates were analysed at 25 °C in methanol-d_4_ using Bruker Ascend III HD 500 MHz NMR spectrometer (Bruker BioSpin GmbH-Rheinstetten, Germany). Standard 1D (^1^H, ^13^C) and 2D (gCOSY, TROESY, gHSQC, gHMBC) pulse programs were used for data acquisition. NMR data was processed using Topspin 3.2 pl7.

#### 3.3.4. Antioxidant Activity

Antioxidant activity of CE, PE, F1 and F4–F6, such as reducing power and DPPH radical-scavenging activity, was determined according to the methods described by Oleszek and Kozachok [51]. Briefly, tested samples were dissolved in methanol in the range of concentrations from 0 to 1500 μg mL^−1^. For reducing power analysis, phosphate buffer (2.5 mL, 0.2 M and pH 6.6) and potassium ferricyanide [K_3_Fe(CN)_6_] (2.5 mL, 1%, *w*/*v*) were adjusted to 1 mL of the solution of tested samples. Next, the samples were incubated at 50°C for 30 min., after which trichloroacetic acid (TCA); (2.5 mL, 10%, w/v) was added. The obtained solutions (2.5 mL) were mixed with deionised water (2.5 mL) and ferric chloride (FeCl_3_); (0.5 mL, 0.1%, *w*/*v*). The absorbance was measured at 700 nm. The results were expressed as EC_50_, which was the concentration that gave absorbance equal to 0.5. Ascorbic acid was used as the reference sample.

Radical-scavenging activity was determined by the reaction of the solutions of the samples (3 mL) with 1,1-diphenyl-2-picrylhydrazyl (DPPH) radical (1 mL, 0.1 mM). Purple radical solution was discoloured and the colour change was stated by measurement of the absorbance at 517 nm. DPPH radical-scavenging activity was calculated according to the following formula:

%Inhibition = [(*A*_0_ − *A*_1_)/*A*_0_] × 100 (1)

where: *A*_0_ was the absorbance for the reference sample (DPPH solution) and *A*_1_ was the absorbance for the tested sample. The results were presented as IC_50_, which was the concentration, which corresponded to 50% of inhibition.

The values of EC_50_ and IC_50_ were expressed as means ± standard deviations from three replicates. The significance of differences between tested samples were evaluated by the Tukey post-hoc test at *p* < 0.05.

#### 3.3.5. Antifungal Activity

Antifungal activity analysis was performed using 96-well MT2 microplates (Biolog^®^, Hayward, CA, USA) according to the method of Frąc et al. [10] with modifications. The aqueous solutions of tested samples were prepared in the concentrations of 0, 5, 50, 100 and 500 μg mL^−1^. One hundred microliters of each solution was added to each well inoculated previously with 50 μL (containing ca. 5–17.5 × 10^4^ spores) of the fungal mycelium suspended in filamentous fungi inoculating fluid (IF-FF) (Biolog^®^, Hayward, CA, USA). Before inoculation, the suspension was standardized for each isolates into 75% transmittance (1–3.5 × 10^6^ spores/mL, depending on the fungal strain). Wells filled with each tested solution or water with the IF-FF fluid without fungus were used as the controls. Three experimental replicates for each test were used. The MT2 plates were inoculated with 100 µL of inoculum per well. The plates were incubated at 26 °C for 8 days. The absorbance was measured every day at the wavelength of 490 nm as mitochondrial activity (substrate utilization) and 750 nm as mycelial growth (growth pattern) using microstation (Biolog^®^). The results were expressed as the ratio of absorbance for tested samples with fungi and absorbance for adequate control (sample alone, without fungi); (A_1_/A_0_). Moreover, the ratio of absorbance at 490 nm and at 750 nm (A490/A750), indicating the metabolic intensity compared to biomass production, were analysed to better explain the metabolisms of tested fungi [36].

For data analysis, the mean value of all days was taken and expressed as means ± standard deviations from 24 replicates (3 replicates for each of 8 days). The significance of differences between tested formulations and control were evaluated by Tukey’s post-hoc test at *p* < 0.05.

## Figures and Tables

**Table 1:** Quantification of compounds in crude and purified extracts, as well as selected fractions of apple pomace.

R_t_ (min)	Compound	MW (g mol^−1^)	% *w*/*w* (Relative)					
			^1^ CE	PE	F1	F4	F5	F6
3.77	Pinnatifidanoside D (vomifoliol-9-O-[β-D-Xyl(1→6)-β-D-Glc])	518	0.11 (14)	1.23 (16)	5.9 (100)	-	-	-
6.29	Hyperoside (Q-3-O-β-D-Gal)	464	0.16 (21)	1.55 (20)	-	-	33.4 (43)	1.91 (3)
6.33	Rutin (Q-3-O-α-L-Rha(1→6)-β-D-Glc)	610	*	*	-	2.3(5)	-	-
6.64	Isoquercetin (Q-3-O-β-D-Glc)	464	0.02 (3)	0.25 (3)	-	-	5.2 (7)	-
7.20	Reynoutrin (Q-3-O-β-D-Xyl)	434	0.05 (6)	0.52 (7)	-	-	-	20.17 (32)
7.53	Q-3-O-pentosyl	434	-	0.05 (1)	-	-	-	1.82 (3)
7.91	Avicularin (Q-3-O-α-L-Ara)	434	0.1 (13)	1.02 (13)	-	-	3.5 (5)	34.10 (53)
8.19	Q-3-O-pentosyl	434	0.02 (2)	0.16 (2)	-	-	-	5.86 (9)
8.47	Quercitrin (Q-3-O-α-L-Rha)	448	0.15 (19)	1.58 (20)	-	-	34.9 (45)	-
10.31	Phloridzin (phloretin-2′-O-β-D-Glc)	436	0.17 (22)	1.29 (17)	-	44.7 (95)	-	-
12.53	Quercetin	302	*	0.09 (1)	-	-	-	-
Total, % *w*/*w*			0.77	7.75	5.9	47.00	77.04	63.86

^1^ CE—crude extract; PE—purified extract; F—LH20 fractions; Q—quercetin; *—traces; R_t_—retention time; MW—molecular weight.

**Table 2:** Antioxidant activity of tested samples.

Sample	Reducing Power EC_50_ (μg mL^−1^)	Radical-Scavenging Activity IC_50_ (μg mL^−1^)
CE	>1500	>1500
PE	298.33 ± 5.84	444.65 ± 10.57
PF	>1500	>1500
F1	460.60 ± 28.84	1117.21 ± 59.10
F4	137.38 ± 1.61	188.54 ± 7.95
F5	100.83 ± 1.62	105.92 ± 1.23
F6	93.94 ± 2.68	107.22 ± 1.77
Ascorbic acid	27.82 ± 0.07	73.61 ± 6.35

^1^ CE—crude extract; PE—purified extract; PF—polar fraction; F—LH-20 subfraction.

**Table 3:** The ratio of absorbance at 490 nm to absorbance at 750 nm (A490/A750).

Tested Sample	Concentration (µL mL^−1^)	A490/A750			
		*N. fischeri*	*F. oxysporum*	*Botrytis sp.*	*P. setifera*
Crude extract	0	0.93	0.96	0.89	0.89
	5	0.97	1.05	1.09	0.91
	50	1.00	1.07	1.10	0.80
	100	0.99	1.07	1.08	0.91
	500	1.04	1.15	1.14	0.74
Purified extract	0	0.93	0.96	0.89	0.89
	5	1.02	1.07	1.06	1.07
	50	0.99	1.03	1.06	0.92
	100	0.98	1.01	1.11	0.71
	500	0.95	1.01	1.03	0.52
Polar fraction of the extract	0	0.93	0.96	0.89	0.89
	5	0.96	1.02	1.02	1.01
	50	1.00	1.06	1.03	0.94
	100	0.99	1.05	1.03	0.90
	500	1.04	1.10	1.04	0.94
Fraction 1	0	0.93	0.96	0.89	0.89
	5	0.96	1.00	0.98	0.67
	50	0.98	1.02	1.01	0.59
	100	0.97	1.01	1.02	0.75
	500	0.94	0.97	0.95	0.59
Fraction 4	0	0.93	0.96	0.89	0.89
	5	0.95	0.97	1.04	0.67
	50	0.93	1.04	0.97	0.78
	100	0.98	1.13	1.01	0.96
	500	1.03	1.44	1.20	1.26
Fraction 5	0	0.93	0.96	0.89	0.89
	5	0.93	0.95	0.95	0.58
	50	0.93	0.94	0.93	0.62
	100	0.93	0.95	0.94	0.75
	500	1.01	1.02	1.02	0.89
Fraction 6	0	0.93	0.96	0.89	0.89
	5	1.01	1.07	1.09	0.69
	50	1.04	1.13	1.11	0.85
	100	1.02	1.12	1.05	0.79
	500	1.00	1.06	1.10	0.82

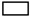
—A490/A750 < 0.75, 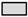
—0.75 ≤ A490/A750 ≤ 0.95, 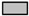
—0.95 ≤ A490/A750 < 1.05, 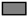
—1.05 ≤ A490/A750 ≤ 1.10, 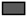
—1.10 < A490/A750 ≤ 1.20, 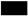
—1.20 < A490/A750.

## Supplementary Materials

The following are available online at https://www.mdpi.com/2072-6651/11/6/361/s1, Figure S1: LC-DAD and MS/ES- chromatograms of crude extract from apple pomace, Figure S2: LC-DAD and MS/ES- chromatograms of purified extract from apple pomace, Figure S3: LC-DAD and MS/ES- chromatograms of fraction 1 from apple pomace, Figure S4: LC-DAD and MS/ES- chromatograms of fraction 4 from apple pomace; Figure S5: LC-DAD and MS/ES- chromatograms of fraction 5 from apple pomace, Figure S6: LC-DAD and MS/ES- chromatograms of fraction 6 from apple pomace, Figure S7: ^1^H and ^13^C NMR data of pinnatifidanoside D. Figure S8: ^1^H NMR (500 MHz) spectrum of pinnatifidanoside D, in MeOH-d_4_, 25 °C, Figure S9: ^13^C NMR (125 MHz) spectrum of pinnatifidanoside D, in MeOH-d_4_, 25 °C, Figure S10: ^1^H-^1^H 2D COSY NMR (500 MHz) spectrum of pinnatifidanoside D, in MeOH-d_4_, 25 °C, Figure S11: ^1^H-^1^H 2D NOESY NMR (500 MHz) spectrum of pinnatifidanoside D, in MeOH-d_4_, 25 °C, Figure S12: ^1^H-^13^C HSQC NMR (500 MHz) spectrum of pinnatifidanoside D, in MeOH-d_4_, 25 °C, Figure S13: ^1^H-^13^C H2BC NMR (500 MHz) spectrum of pinnatifidanoside D, in MeOH-d_4_, 25 °C, Figure S14: ^1^H-^13^C HSQC-TOCSY NMR (500 MHz) spectrum of pinnatifidanoside D, in MeOH-d_4_, 25 °C, Figure S15: ^1^H-^13^C HMBC NMR (500 MHz) spectrum of pinnatifidanoside D, in MeOH-d_4_, 25 °C. Figure S16: LH-20 chromatogram of plant specific metabolites fraction from apple pomace.
