# Supplementary material for: Phytochemicals of Apple Pomace as Prospect Bio-Fungicide Agents against Mycotoxigenic Fungal Species—In Vitro Experiments
Source: Toxins (Basel). 2019 Jun 20;11(6):361. doi: 10.3390/toxins11060361 (PMC6628436; doi:10.3390/toxins11060361)
Supplement: Supplementary file 1 [file toxins-11-00361-s001.pdf]

Supplementary Materials: Phytochemicals of Apple Pomace as Prospect Bio-Fungicide Agents against Mycotoxigenic Fungal Species—In Vitro Experiments

Marta Oleszek*, Łukasz Pecio, Solomiia Kozachok, Żaneta Lachowska-Filipiuk, Karolina Oszust and Magdalena Frąc

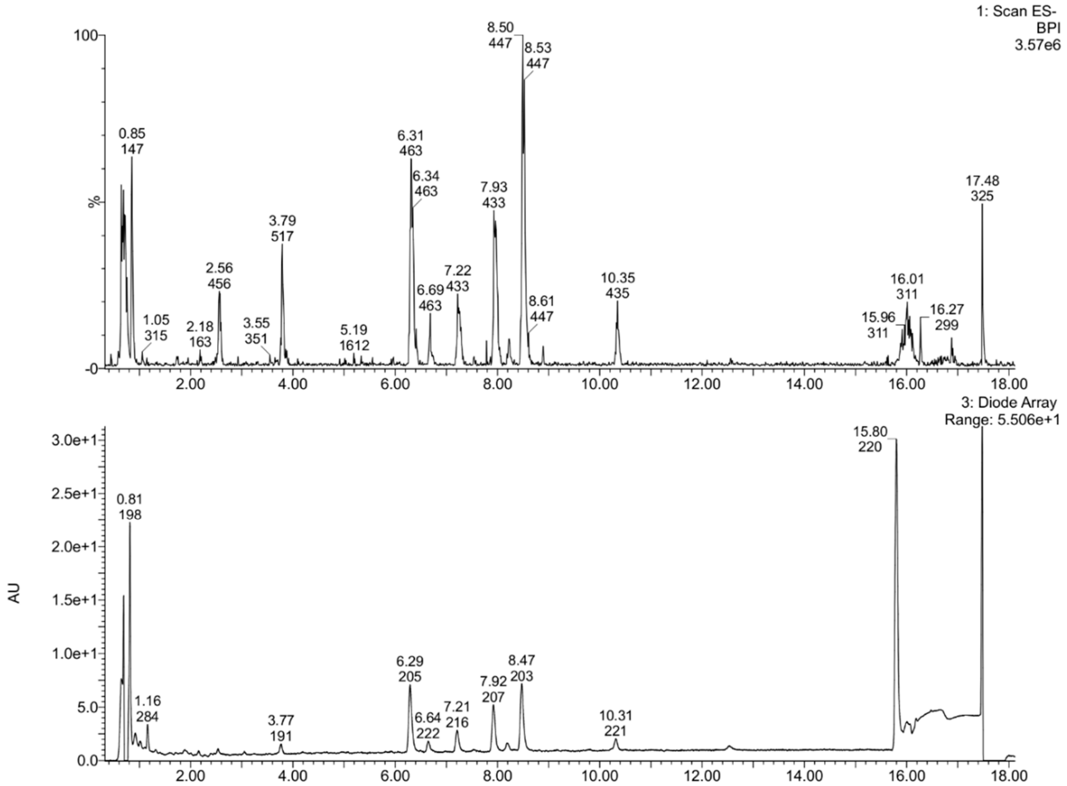

Figure S1. LC-DAD and MS/ES- chromatograms of crude extract from apple pomace.

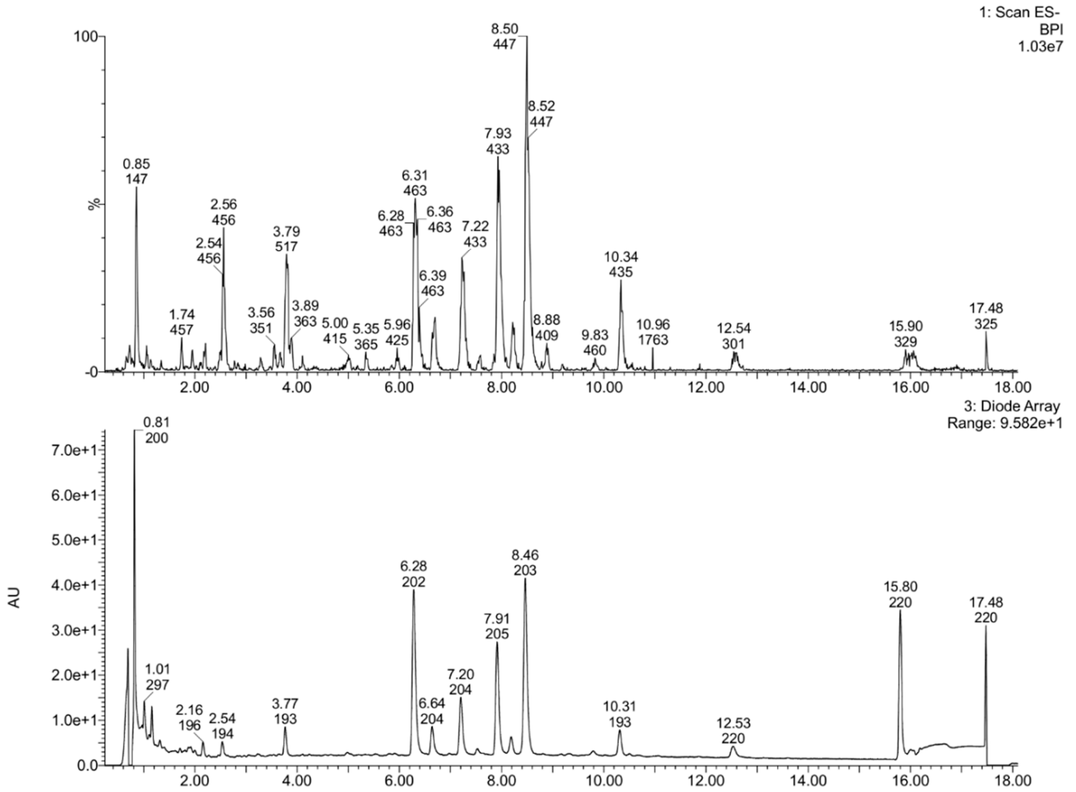

Figure S2. LC-DAD and MS/ES- chromatograms of purified extract from apple pomace.

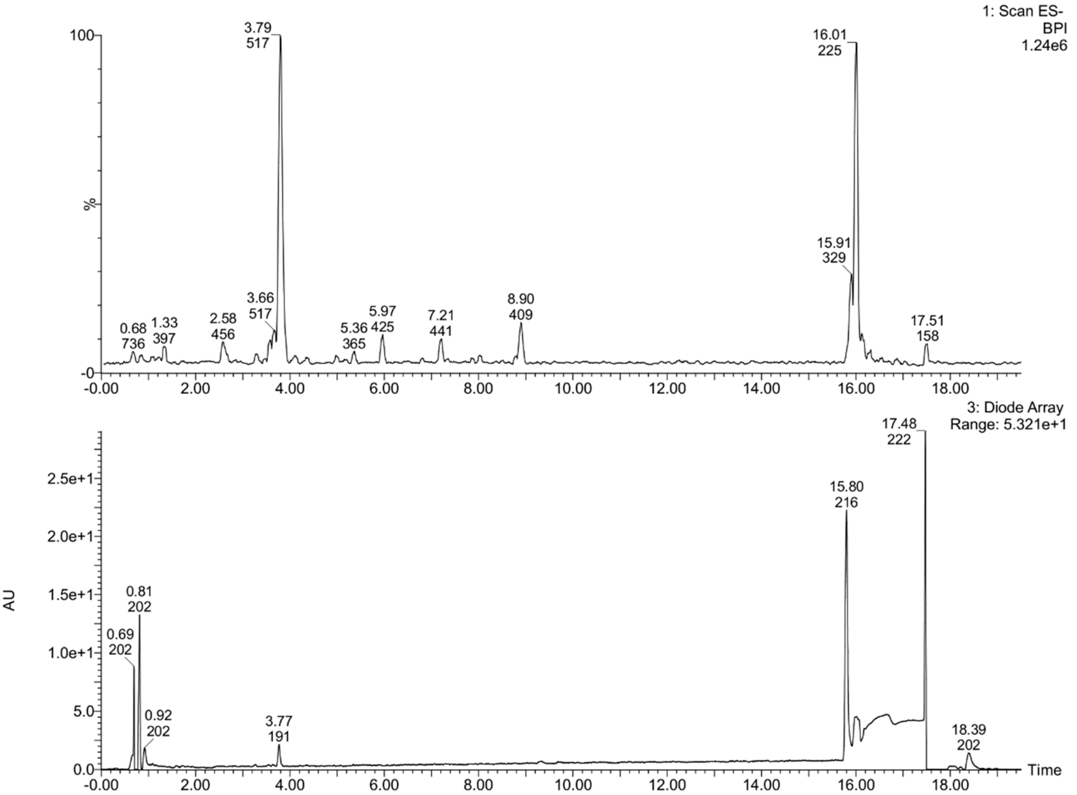

Figure S3. LC-DAD and MS/ES- chromatograms of fraction 1 from apple pomace.

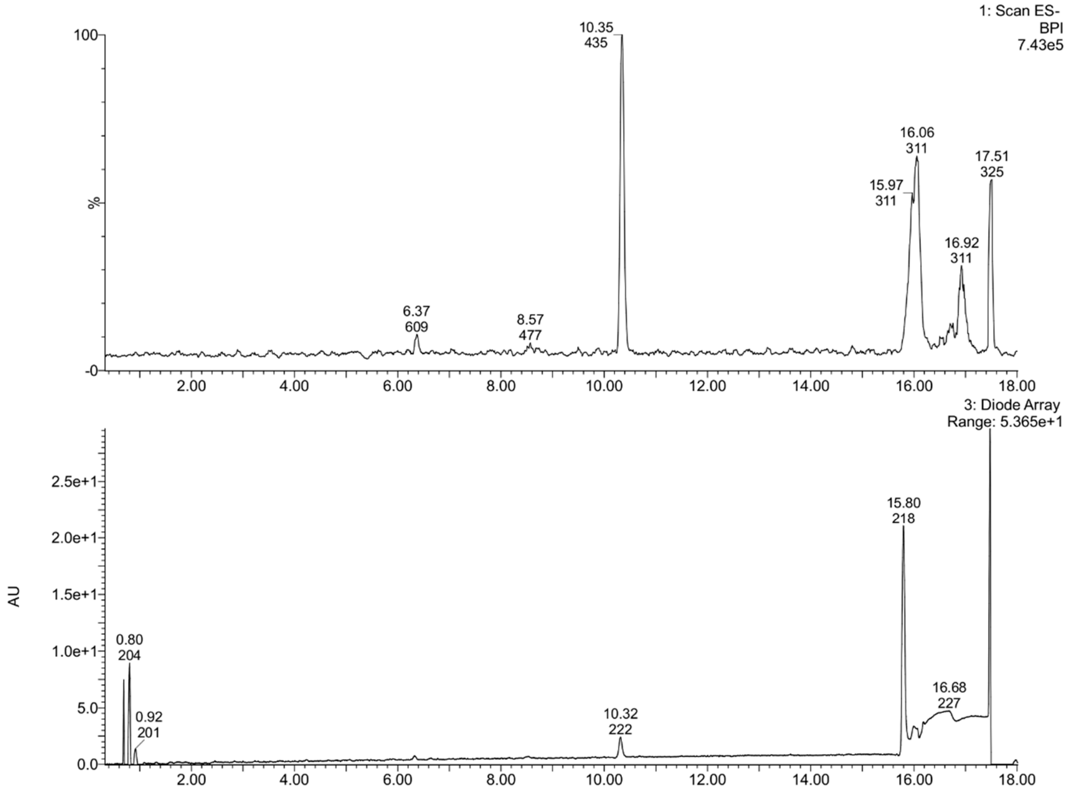

Figure S4. LC-DAD and MS/ES- chromatograms of fraction 4 from apple pomace.

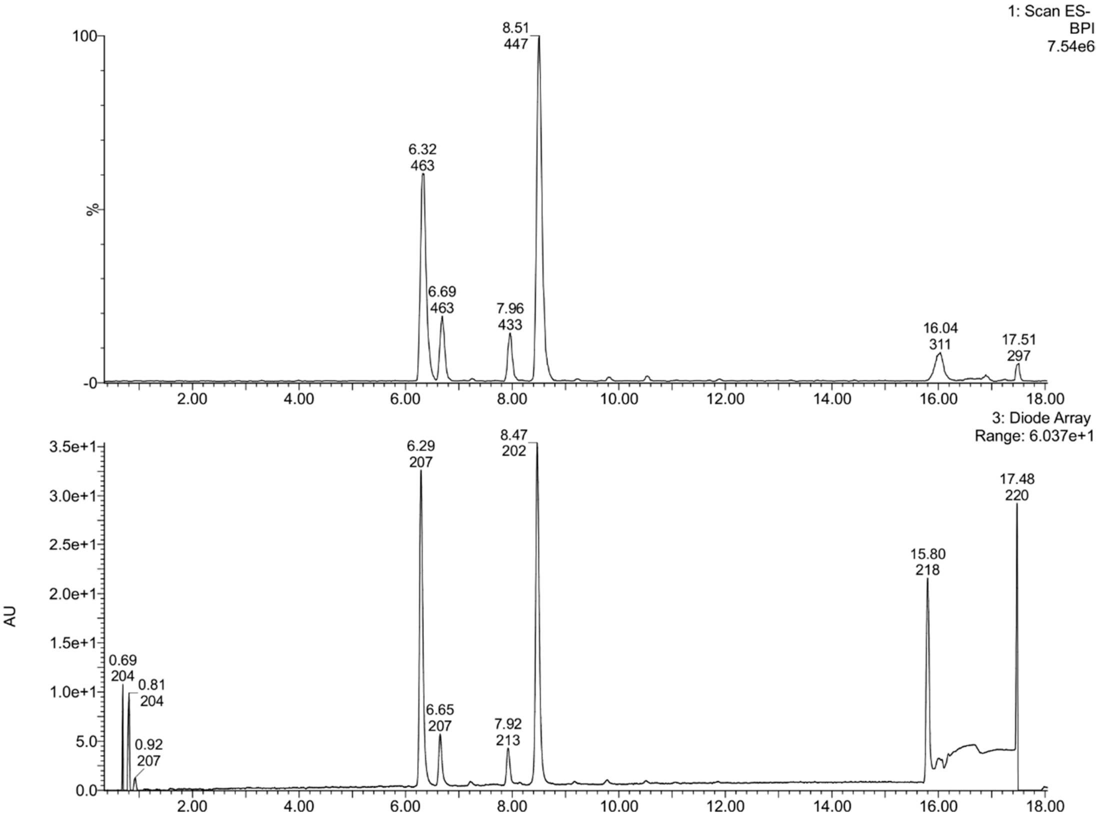

Figure S5. LC-DAD and MS/ES- chromatograms of fraction 5 from apple pomace.

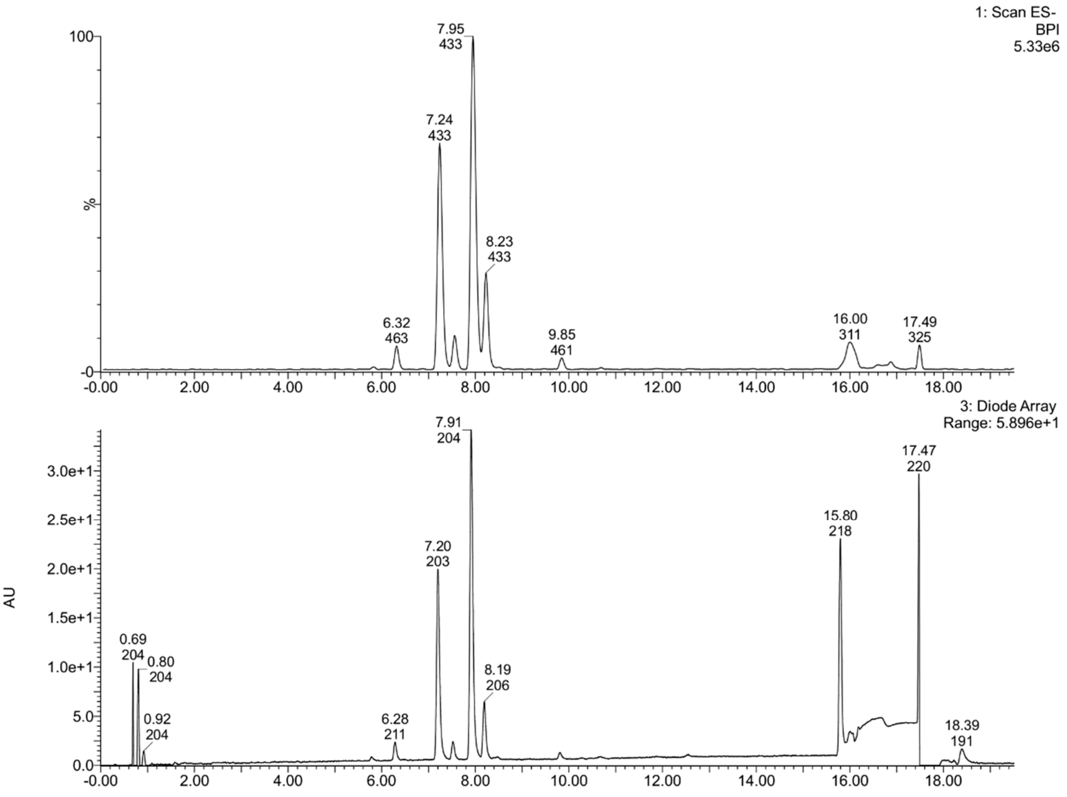

Figure S6. LC-DAD and MS/ES- chromatograms of fraction 6 from apple pomace.

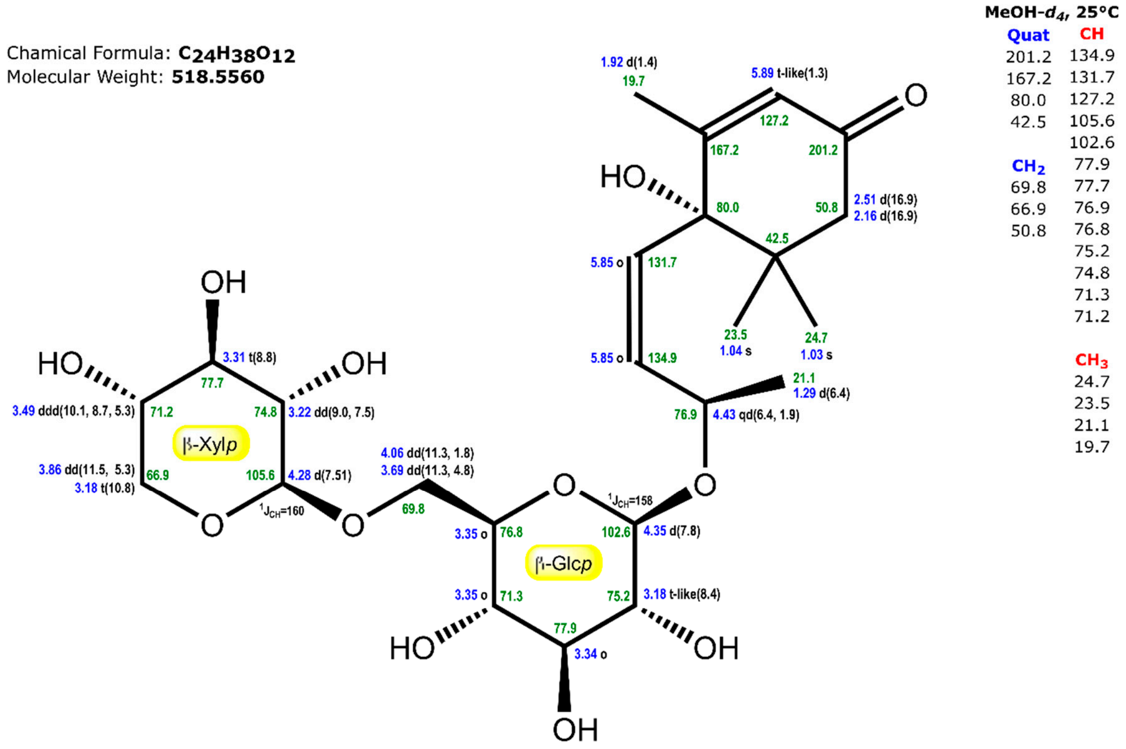

Figure S7. 1H and 13C NMR data of pinnatifidanoside D.

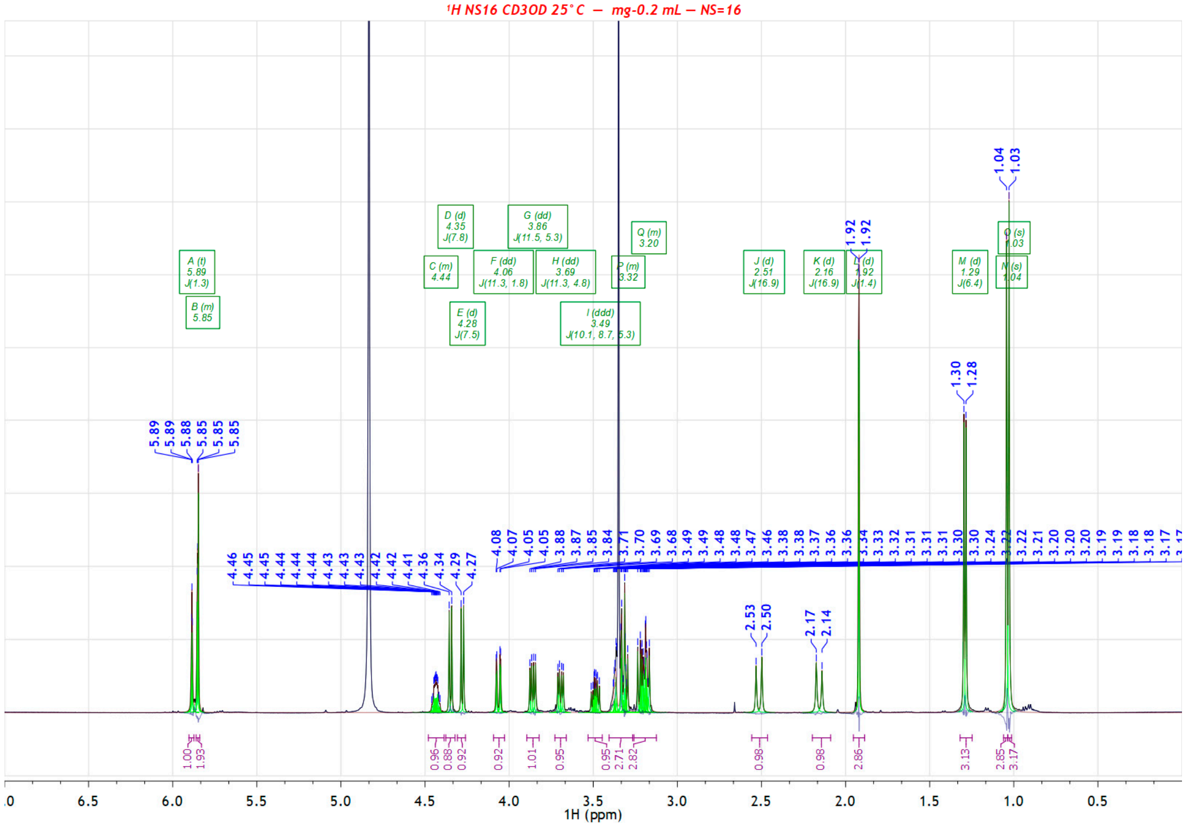

Figure S8. ^{1}H NMR (500 MHz) spectrum of pinnatifidanoside D, in MeOH-d_4, 25 °C.

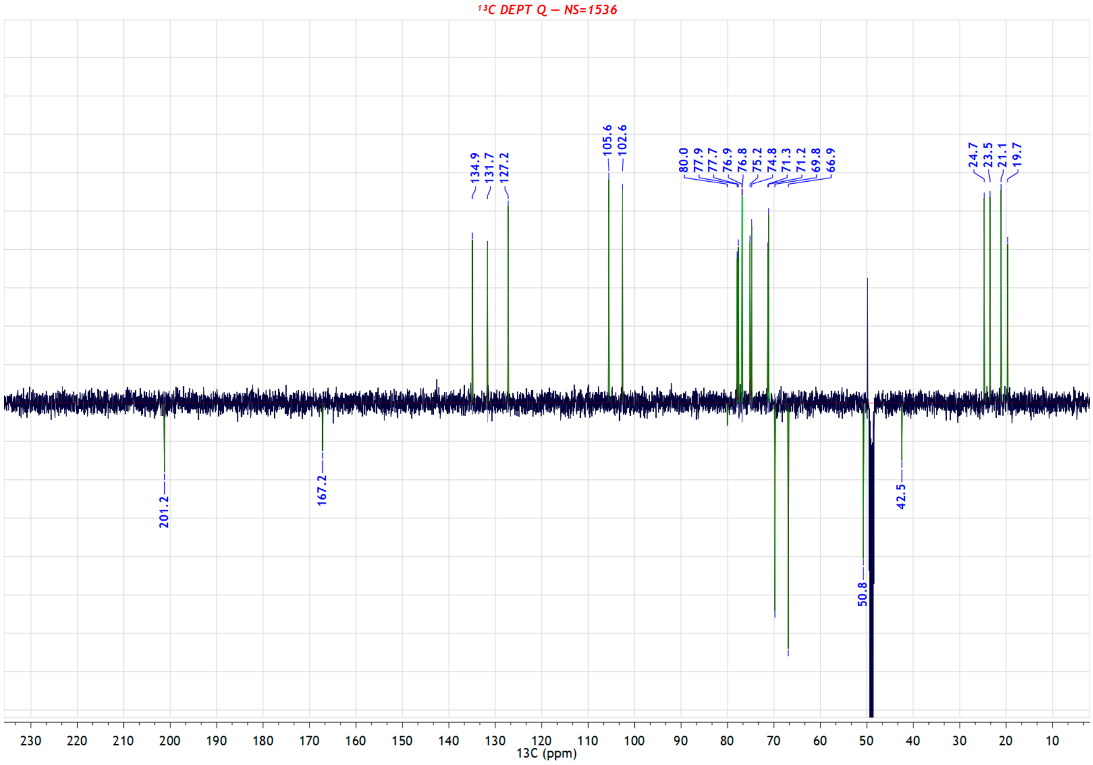

Figure S9. ^{13}C NMR (125 MHz) spectrum of pinnatifidanoside D, in MeOH-d_4, 25 °C.

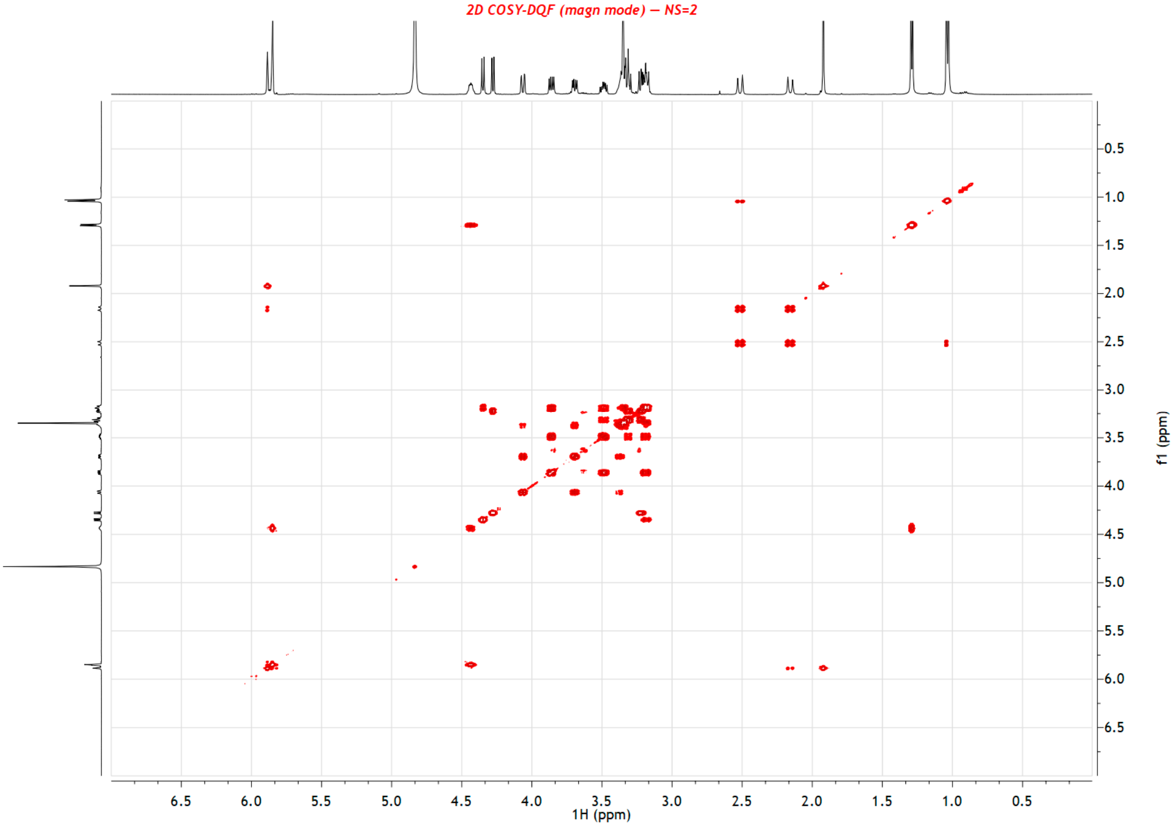

Figure S10. ^{1}H-^{1}H 2D COSY NMR (500 MHz) spectrum of pinnatifidanoside D, in MeOH-d_4, 25 °C.

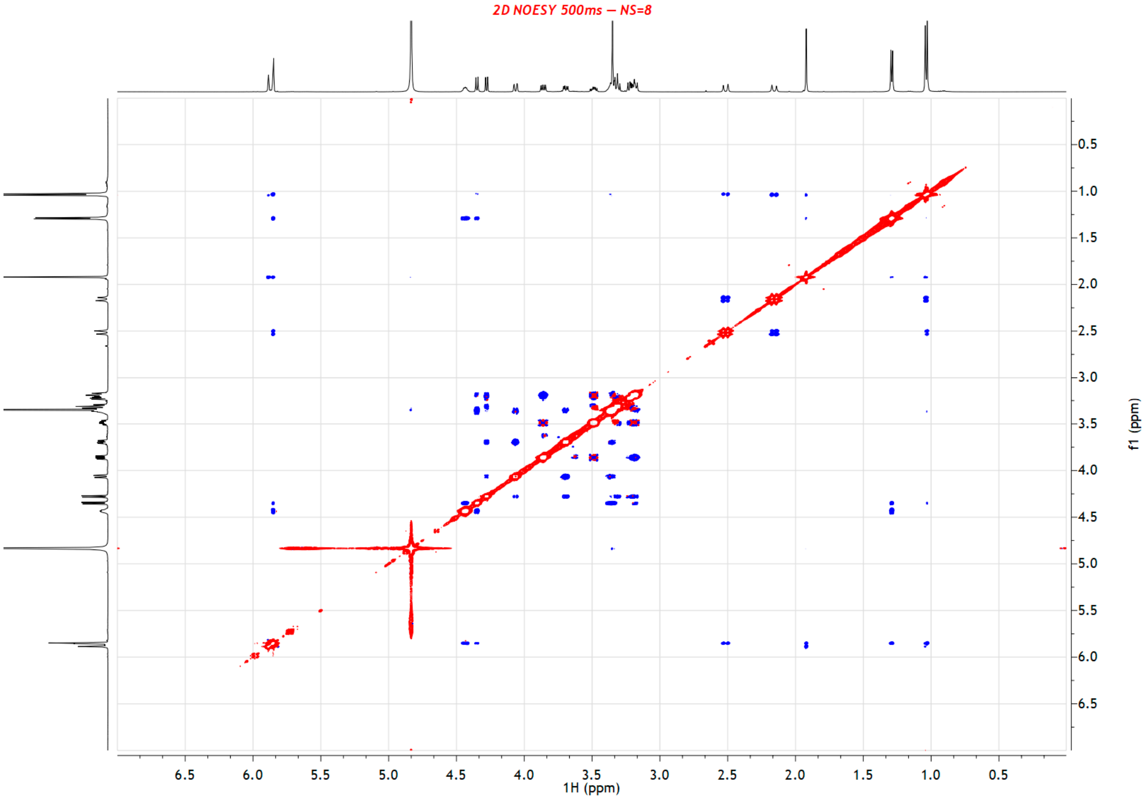

Figure S11. ^{1}H-^{1}H 2D NOESY NMR (500 MHz) spectrum of pinnatifidanoside D, in MeOH-d_4, 25 °C.

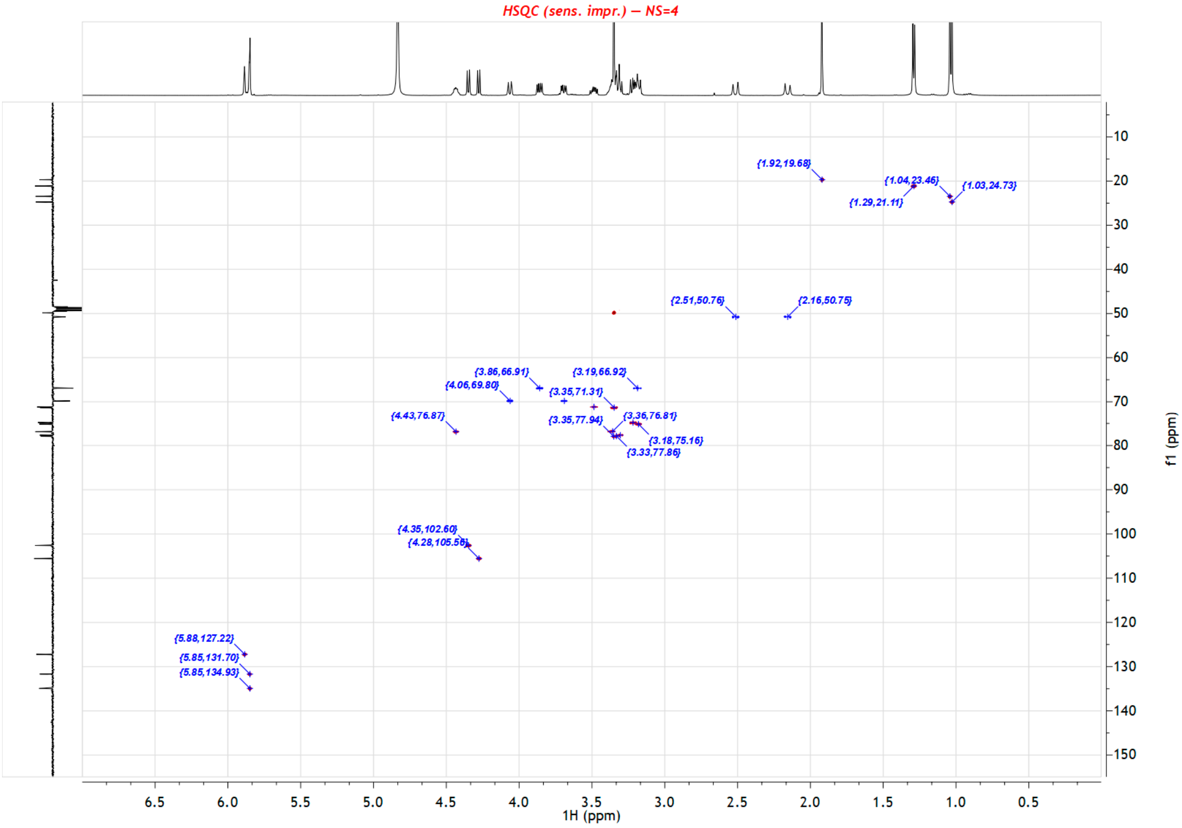

Figure S12. ^1H-^{13}C HSQC NMR (500 MHz) spectrum of pinnatifidanoside D, in MeOH-d_4, 25 °C.

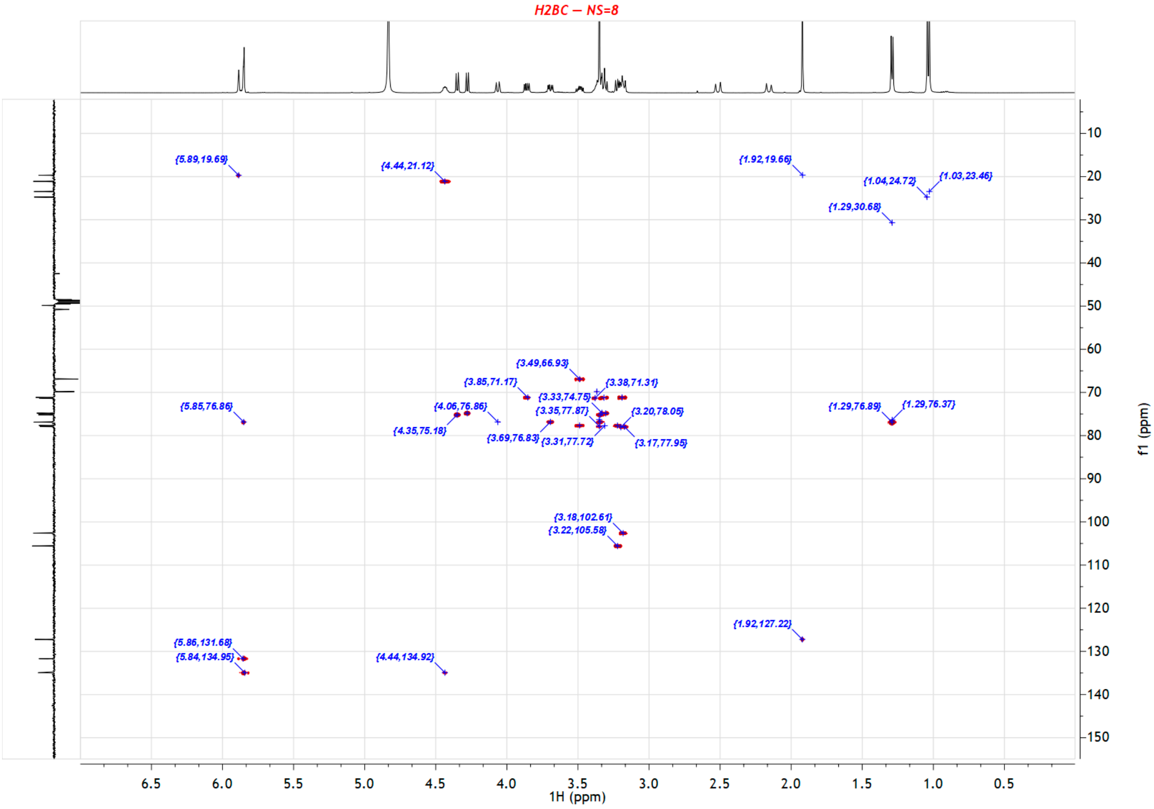

Figure S13. ^1H-^{13}C H2BC NMR (500 MHz) spectrum of pinnatifidanoside D, in MeOH-d_4, 25 °C.

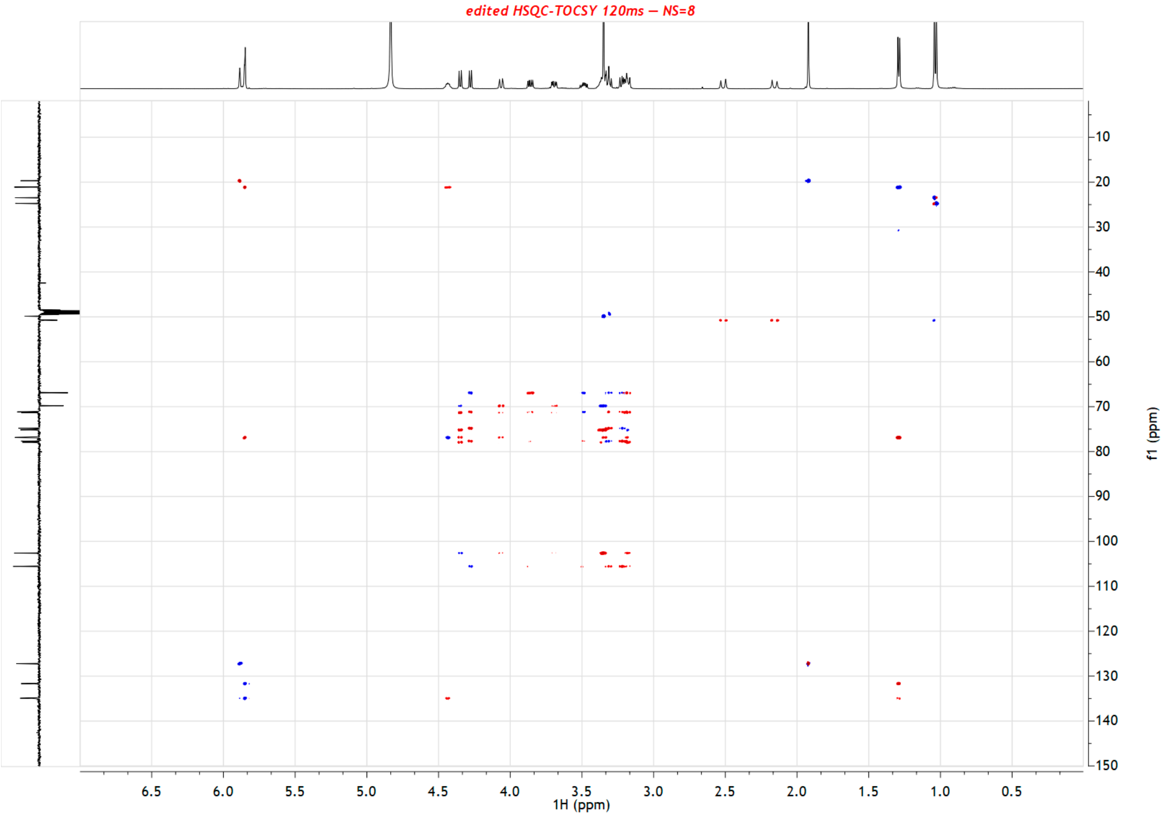

Figure S14. ^{1}H-^{13}C HSQC-TOCSY NMR (500 MHz) spectrum of pinnatifidanoside D, in MeOH-d4, 25 °C.

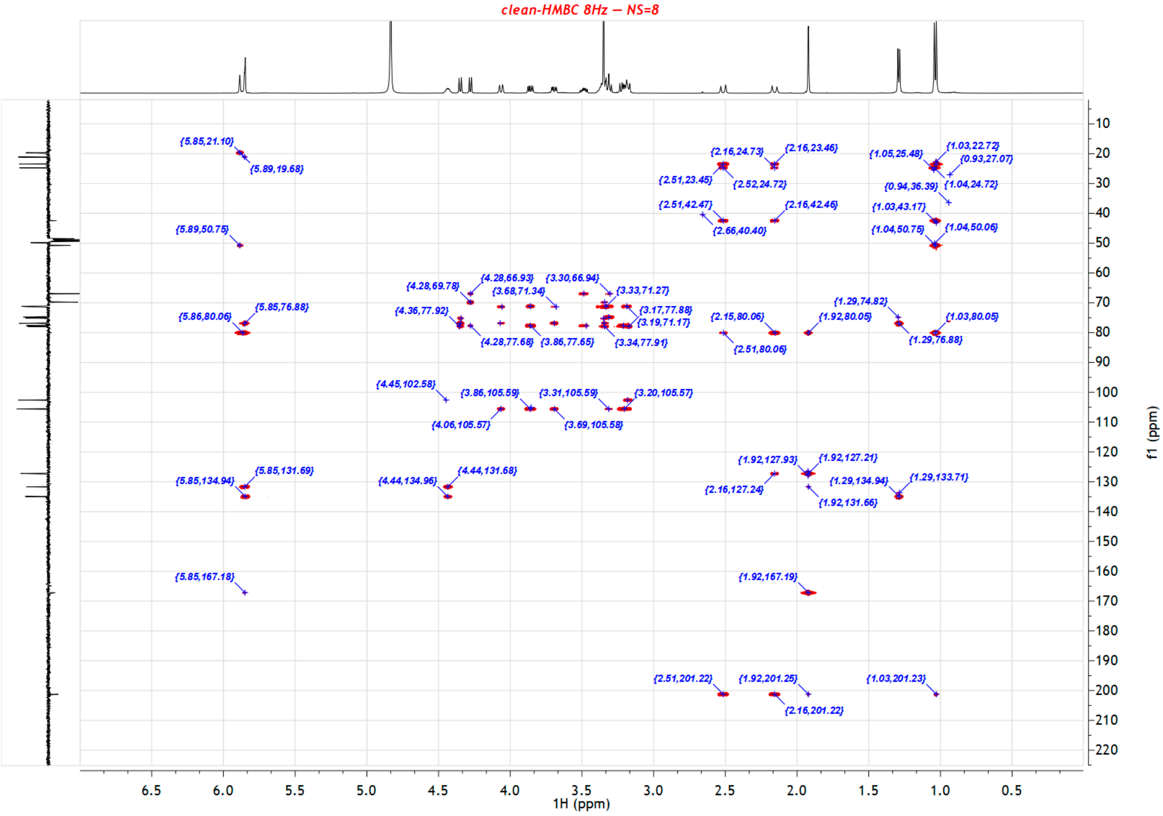

Figure S15. ^{1}H-^{13}C HMBC NMR (500 MHz) spectrum of pinnatifidanoside D, in MeOH-d4, 25 °C.

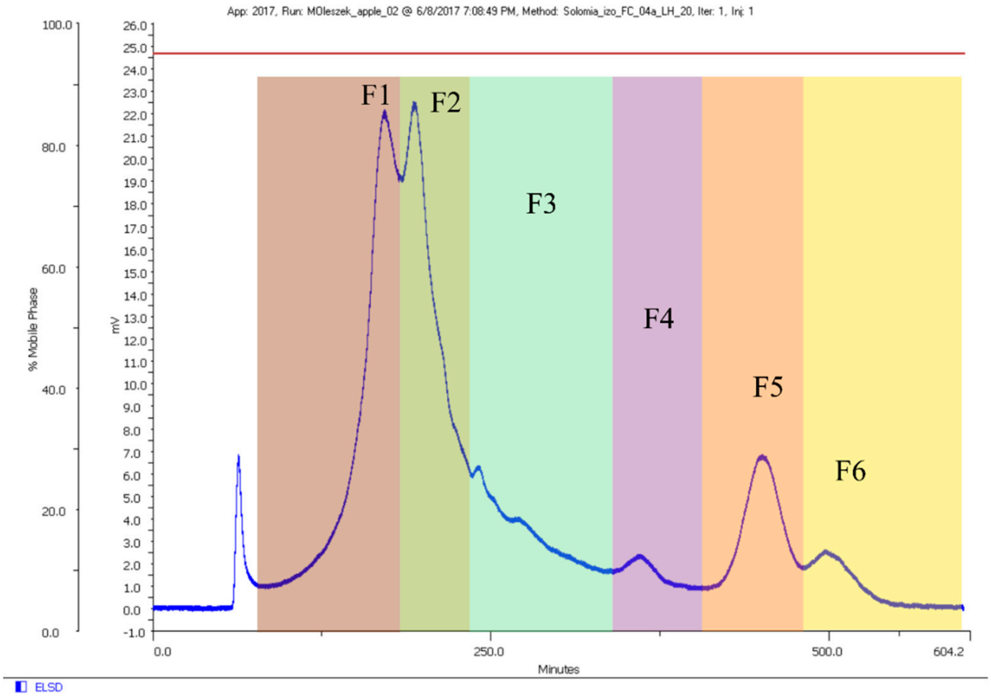

Figure S16. LH-20 chromatogram of plant specific metabolites fraction from apple pomace.
